# Cost-effectiveness of biofire assay for respiratory infection testing: An economic evaluation exploring the inclusion of the costs of antimirobial resistance

**DOI:** 10.1371/journal.pone.0347991

**Published:** 2026-04-27

**Authors:** Raymond Oppong, Raj Gill, Kay Roy

**Affiliations:** 1 Health Economics Unit, University of Birmingham, Birmingham, United Kingdom; 2 The Bloomsbury Surgery, London, United Kingdom; 3 University College Hospital, London, United Kingdom; Mzuzu University Faculty of Information Science and Communications: Mzuzu University Faculty of Science Technology and Innovation, MALAWI

## Abstract

**Background:**

Patients diagnosed with chronic obstructive pulmonary disease (COPD) often face acute symptom exacerbations. Current management approaches lack precision in diagnosis. To address this, the Biofire Film Array Respiratory Panel RP 2.1 (RP 2.1+) test was developed, aiming to swiftly confirm or rule out common respiratory viral and bacterial infections. This study aims to evaluate the RP 2.1 + test’s cost-effectiveness and explore the potential impact of accounting for the costs of antimicrobial resistance.

**Methods:**

We conducted a model-based cost-utility analysis from a healthcare perspective over 40 years. The design of our model, along with its parameters, was informed by insights from a targeted literature review and expert opinions. To account for antimicrobial resistance (AMR) two approaches were adopted. Including applying penalty points to the cost of antibiotic prescriptions and estimating the potential cost savings from reductions in AMR.

**Results:**

The results indicate that the RP 2.1 + test is cost-saving and more effective. The difference in costs and QALYs between arms were £2762.40 and 0.03 respectively and there is a 70% chance that the RP 2.1 + test is cost-effective. The analysis suggest that potential annual savings from adopting the RP 2.1 + test could range between £118 million and £18.6 billion annually.

**Conclusion:**

The RP 2.1 + test represents a cost-effective use of healthcare resources and would lead to significant savings with respect to AMR. The study has attempted to account for AMR within the economic evaluation and has highlighted areas where further research is needed to account for AMR more precisely in future evaluations.

## Introduction

Chronic obstructive pulmonary disease (COPD) exacerbations is an acute worsening of the COPD disease [1]. Exacerbations are changes in the normal disease “dyspnea, cough, and/or sputum” that might need a change in the patient’s medication [2]. In primary care, over 70% [3–5] presenting with acute exacerbation of chronic obstructive pulmonary disease (AECOPD) receive antibiotics, often requiring frequent courses with subsequent hospitalisation [6]. NHS long-term plan focuses on the need to perform diagnostics earlier [7] and ensure accurate treatment plans for respiratory disease with more initial care undertaken in community settings to diagnose and treat flare ups like AECOPD [8,9].

Antibiotics are usually used as first line treatment for exacerbations, and a major challenge in managing patients with exacerbations of chronic obstructive pulmonary disease (COPD) is determining which patients will benefit from antibiotic treatment. It is often impractical for clinicians to order specialist microbiological or other investigations for every patient presenting with symptoms of acute respiratory infections. Instead, clinicians routinely rely on the patient’s medical history and a physical clinical assessment. However, clinical assessment alone has poor predictive value. Consequently, antibiotics are often prescribed to those who are unlikely to benefit, contributing to the development of antimicrobial resistance [10–12].

The Biofire FilmArray Respiratory Panel RP 2.1 + , which detects viruses and can be used to rapidly rule-in or rule-out common types of respiratory viral and bacterial infection without the need for specialist laboratory support and expertise. However, there is a lack of high-quality evidence on the clinical and cost-effectiveness of this test specifically in the community and primary care settings.

The aim of this health economics study is to conduct a preliminary analysis of the potential cost-effectiveness of the Biofire FilmArray Respiratory Panel RP 2.1+ in patients experiencing exacerbations of COPD in community setting. The economic analysis will also seek to explore the implications of accounting for antimicrobial resistance in economic evaluation studies.

## Materials and methods

A model-based economic evaluation was carried out to evaluate the economic and health-related outcomes of employing the Biofire FilmArray Respiratory Panel RP 2.1 + test in guiding treatment for COPD exacerbations. This was compared with the standard treatment practices for COPD exacerbations in the United Kingdom [13]. The current treatment guidelines in UK recommend that patients with moderate exacerbations should be treated with corticosteroids steroid, antibiotics or both, while patients with severe exacerbations should be hospitalised [14]. In this arm, initial treatment decisions, including whether to prescribe antibiotics, were based on routine clinical assessment and judgement rather than rapid molecular diagnostic testing. In the intervention arm, the Biofire FilmArray Respiratory Panel RP 2.1 + test was utilized, ensuring that only patients with bacterial-caused exacerbations received antibiotic treatment. Conversely, in the control group representing current practice, treatment decisions were based on clinical judgment.

### Literature review and existing modelling approaches

A targeted literature search was conducted using PubMed and google scholar, with search terms relating to the disease. Searches were limited to papers published between 2010 and 2023 to ensure greater relevance to the current clinical landscape. The aim of this targeted review was to identify studies that can inform the structure and inputs of a model to assess the cost-effectiveness of Biofire FilmArray Respiratory Panel RP 2.1 + test.

### Economic evaluation

The analysis compared costs and outcomes of the intervention and current practices from a UK National Healthcare Service and personal social services perspective [15]. Costs were expressed in British pound sterling and outcomes were expressed in quality adjusted life years (QALY), a measure of health status that combines quantity and quality of life lived by a person [16]. The results of the analysis were presented using incremental cost-effectiveness ratio (ICER) [13,15]. The ICER was compared to the £20,000 willingness to pay threshold per QALY gain in line with the recommendations in UK [17].

### Type of model

The COPD exacerbation model combined a decision tree to model short-term outcomes and a Markov model to model the longer-term outcomes given the chronic nature of COPD. The model was adapted from a previous study [18]. Pathways of the current model were agreed after discussions between the economist and clinical experts with experience in COPD management and respiratory infection diagnosis. These discussions were used to validate the plausibility of pathways, assumptions, and usual-care comparators in UK practice. The expert input comprised advisory discussions to inform model structure and assumptions. All modelling were conducted in Microsoft Excel 2016 (Microsoft Corporation, Redmond, WA, USA).

The decision tree was built to assess the short-time costs and outcomes in line with the follow-up period commonly used in COPD exacerbations studies [19–21]. The model pathways for the decision tree have been presented in S1 Fig and S2 Fig where patients in the intervention and current practice arms have identical pathways. In both arms, a proportion of patients presenting with COPD exacerbations could be prescribed with antibiotics while the other patients can be prescribed with steroids and antibiotics. Proportion of patients would improve and fully recover, while the condition deteriorated for the rest of the patients that were on antibiotics. Some of the patients whose condition deteriorated fully recover after a second treatment. Patients that did not recover following third treatment required hospital admission. The admitted patients either fully recovered, partially recovered, or died.

Intermediate outcomes from the decision tree were used as some input parameters for the Markov model. Patients that recovered after first treatment were considered to have mild exacerbations, those who recovered after the second or more treatments were considered to have moderate exacerbations while those that needed hospitalisations were considered to have severe exacerbations [18]. The Markov model was developed to assess costs and benefits for lifetime events and was run for 10,000 patients aged 60–100 years who had COPD exacerbations with three different stages: GOLD Stage II, Stage III, and Stage IV in line with a previous modelling that has been conducted in this area S3 Fig [18].

### Model input parameters

#### Resource use and unit costs.

Data on resource use were sourced from a previous study [18]. The resources were valued using 2022 average unit costs of resources that were sourced from various published unit costs in the UK (Table 1) [22–24]. The 2022 unit costs of spirometry in both primary and secondary care were not available and was estimated by inflating a previously reported cost using an online inflation calculator provided by the Bank of England [25].

**Table 1:** Model costs.

Item	Unit cost	Standard Error	Distribution	Source
Prescription costs	£29.00	5.80	Gamma	BNF 2023 [22]
Phone call	£8.80	1.76	Gamma	PSSRU 2022 [23]
Patient contact	£4.46	0.89	Gamma	PSSRU 2022 [23]
Home visit	£115.87	23.17	Gamma	NHS reference costs 2022 [24]
GP consultation	£53.36	10.67	Gamma	PSSRU 2022 [23]
A&E without admission	£499.00	99.80	Gamma	NHS reference costs 2022 [24]
COPD hospital stay	£1,930.01	386	Gamma	NHS reference costs 2022 [24]
Community nurse’s follow-up	£122.00	24.40	Gamma	PSSRU 2022 [23]
Outpatients’ follow-up	£50.72	10.14	Gamma	PSSRU 2022 [23]
RP2.1 test cost	£94.00	24.00	Gamma	Biofire Diagnostics 2023[26]
Antibiotic course	£1.84	0.37	Gamma	BNF 2023 [22]
Steroid course	£0.70	0.14	Gamma	BNF 2023 [22]
Alternative antibiotics	£1.01	0.20	Gamma	BNF 2023 [22]
Secondary care follow-up	£76.08	15.22	Gamma	NHS reference costs 2022 [24]
Primary care follow -up	£122.00	24.40	Gamma	NHS reference costs 2022 [24]
Secondary care spirometry	£64.56	12.91	Gamma	Abel et al, inflated [18]
Primary care spirometry	£22.35	4.47	Gamma	Abel et al, inflated [18]
Influenza vaccination	£8.00	1.60	Gamma	BNF 2023 [22]
Oxygen therapy	£60.93	12.19	Gamma	NHS reference costs 2022 [24]

#### Outcomes.

Utilities for the model were sourced from published studies. Utilities for patients that partially and fully recovered were assumed to be equivalent to the average utilities of patients with stable and moderate exacerbations and were sourced from a UK report [26]. As such, the utilities for patients that partially and fully recovered were estimated by averaging the utilities of patients with GOLD stage II, III, & IV among patients with stable condition and moderate exacerbations respectively [26]. Utilities associated with GOLD stages II-IV were sourced from a systematic review and meta-analysis of health utility values for COPD [27].

#### Probabilities.

Most probabilities were sourced from a published study [18]. The probability of receiving antibiotics in the intervention arm was assumed to be equal to one minus the test sensitivity (1-sensitivity) sourced from Biofire Diagnostics [28].

#### Assumptions.

The following assumptions were made when developing the models and conducting the analysis.

- The probability of receiving antibiotics in the intervention arm is equal to 1 minus test sensitivity.
- There were no deaths associated with fully recovered exacerbations. The only source of death in this state was all cause mortality.

### Analysis

In the decision tree, a rollback calculation was used to estimate the expected outcomes for both strategies. The expected outcomes are calculated by summing the products of the probabilities of various events occurring and the respective outcomes or consequences of those events [13]. The cost of the intervention was factored into the tree only once in the intervention arm depicting the point at which the cost of the test incurred. Payoffs and costs at the end of the tree were rolled back to estimate the average payoffs and costs at beginning of each strategy based on the probabilities of patients passing through the pathways in the tree [13]. The results from the decision tree were not discounted because the timeframe for the model was not more than one year [15].

The Markov model had three-month cycles that were adopted from the states in a previous model. Health states were also adopted from the same model [18]. Health states in a Markov model are mutually exclusive events and the likelihood of being in a health state is not a function of the previous state implying that a patient could only be in one state during each cycle [13]. The patients could move from stable state, mild, to moderate exacerbations. Each of these had three states of GOLD stage II, III, and IV COPD. Finally, the patients could move to the dead state which was an absorbing state. Deaths due to other causes were factored into the model by calculating all-cause mortality based on the 2022 number of deaths and 2022 population estimates for England and Wales 2022 [29,30]. All costs and outcomes were discounted using the half-cycle method at the 3.5% discount rate recommended rate [13,17].

### Sensitivity analysis

Deterministic sensitivity analysis (DSA) and probabilistic sensitivity analysis (PSA) were conducted for the decision tree and Markov models respectively. The DSA was conducted to identify the parameters that could change the base case results for the decision tree. In the DSA, a parameter was varied while keeping the rest of the parameters constant to establish if the change in that parameter would affect the base case results. We also conducted a threshold analysis to determine how expensive the RP 2.1 + test should be for it not to be cost-effective based on the NICE threshold of £20,000 per QALY gained.

The PSA was conducted to assess the uncertainty surrounding the Markov model results. The PSA was based on 10,000 Monte-Carlo simulations that were run based on the distributions assigned to the input parameters (Tables 1–4). The simulations generated 10,000 sets of incremental costs and incremental QALYs that were presented on a cost-effectiveness plane. Cost-effectiveness acceptability curves were generated to present the probability of each strategy being cost-effective across a range of willingness to pay thresholds.

**Table 2:** utilities for death and recover health states.

Health state	Utility value	Distribution	Standard Error	Source
Dead	0	Fixed	N/A*	Drummond et al 2015 [15]
Partially recovered	0.5558	Beta	0.1248	Jordan et al (2016) [27]
Fully recovered	0.6607	Beta	0.1112	Jordan et al (2016) [27]

*Not applicable.

**Table 3:** Utilities for COPD GOLD stages.

Gold stage	Utility value	Distribution	Standard Error	Source
Stage II	0.7820	Beta	0.1564	Moayeri et al (2016) [27]
Stage III	0.7210	Beta	0.1442	Moayeri et al (2016) [27]
Stage IV	0.6240	Beta	0.1248	Moayeri et al (2016) [27]

**Table 4:** Model probabilities.

Pathway	Probability	Distribution	Standard Error
**Intervention arm**			
Probability of getting antibiotics during initial treatment	0.03^26^	Beta	0.0058
Improve after initial antibiotics	0.85^18^	Beta	0.17
Improve after second treatment (ab & AE)	0.90^18^	Beta	0.18
Improve after second treatment (st & ab)	0.87^18^	Beta	0.174
Improve after initial treatment (st & ab)	0.92^18^	Beta	0.184
Improve after second ab	0.85^18^	Beta	0.17
**Control arm**			
Probability of getting antibiotics during initial treatment	0.80^18^	Beta	0.16
Improve after initial antibiotics	0.80^18^	Beta	0.8
Improve after initial treatment (st & ab)	0.89^18^	Beta	0.177
Improve after second treatment (st & ab)	0.87^18^	Beta	0.174
Improve after second ab	0.80^18^	Beta	0.16
Improve after second treatment (ab & AE)	0.90^18^	Beta	0.18
**Both arms**			
Mortality after hospital admission	0.05^18^	Beta	0.0101
Improve after third treatment	0.89^18^	Beta	0.177

Ab stands for antibiotic, st stands for steroids.

### Accounting for antimicrobial resistance

Assessing the economic impact of antibiotic resistance, is a complex task, compounded by the scarcity of available data. There is currently no consensus on how to achieve this. As a result, we adopted two different approaches.

#### Approach 1.

The first followed an approach outlined by a previous study [31]. This analysis assumed a direct relationship between the frequency of antibiotic prescriptions and the increase in resistance levels. This correlation is supported by evidence from previous research [10,32]. This allowed us to infer a preliminary cost figure for the consequences of resistance.

Our study incorporated data from a recent, extensive review on antimicrobial resistance. This review estimated that, over a 35-year period, antimicrobial resistance could lead to a total global GDP loss of about $100 trillion. This translates to an average annual economic impact of approximately $2.8 trillion, equivalent to around £2.2 trillion.

We then obtained data on the number of antibiotic prescriptions from a study which suggests that consumption of antibiotics is 14.1 Defined Daily Doses (DDDs) per 1000 population per day equating to 40.1 billion DDD per year [33]. Assuming a standard prescription lasts for 5 days, then the number of prescriptions per year would equate to approximately 8 billion annually. This would result in a cost of resistance of $356.96 (£285.84) per prescription. The yearly exchange rates (0.800769) for 2023 were used in this study to convert USD to GBP [34].

An antibiotic resistance modulating factor (RMf) was then added to the cost value in recognition of the fact that the cost is not solely due to human consumption [35]. After applying the exchange rates and RMf, the cost of antimicrobial resistance per prescription obtained was £105.76. This cost was then added to the costs associated with antibiotics in our model. Sensitivity analysis was carried out using values obtained from a study was that conducted by Shrestha et al. 2018 and colleagues [35]. For this study we considered the cost of AMR associated with broad spectrum and narrow spectrum antibiotics.

#### Approach 2.

The second approach involved modelling AMR over time and looking at the effects a reduction in the number of antibiotic prescriptions would have on the development of antimicrobial resistance over time. We obtained data on AMR rates between 2023 and 2040 from a previous study. Details of the methodology for predicting the development of AMR over time has been published elsewhere [36]. The predicted AMR rates used in this study was for Methicillin-resistant *Staphylococcus aureus* (MRSA) and Penicillin-resistant *Streptococcus pneumoniae*. These pathogens were chosen as a proxy for AMR and also due to the availability of data.

The next step was to estimate the impact of the Respiratory 2.1 (RP2.1) Panel (RP2.1) test on the development of AMR. To do this, we assumed a relationship to show how a change in the consumption of antibiotics because of the RP 2.1 + test would lead to a change in the levels of antimicrobial resistance. We assumed that the relationship between antibiotic consumption and the development of AMR was constant over time. In essence we assumed that the reductions in AMR because of the RP2.1 + test was constant over time. Due to the uncertainty and the fact that the relationship between antibiotic consumption and the development of AMR is heterogenous across pathogens, drug classes and settings, we assumed that this could be anywhere between 1% and 25% and therefore conducted the analysis based on 4 different values (1%, 5%, 10% and 25%) to reflect this uncertainty. Previous studies have shown that antibiotic consumption explains a modest but variable proportion of resistance patterns [37,38].

The next step was to apply the estimated cost of AMR which was obtained from the O’Neil study [39] ($2.8 (£2.2) trillion per year) and apply to the predictions of AMR in both the RP2.1 + test scenarios and the current care scenario. We used 2023 as a base year. i.e., we assumed that the annual cost of AMR in 2023 was $2.8 (£2.2) trillion per year. The main assumption that was made here was that the costs would increase or decrease in line with the AMR predictions. Since the cost of AMR is not solely due to human consumption, we applied a RMf to the estimated cost of resistance [35]. After applying the RMf, a recent study [31] estimated that acute exacerbations of COPD accounts for 7.5% of all prescriptions. It was therefore assumed in our analysis that the AMR costs associated with COPD exacerbations was 7.5% of the resulting costs. The difference in cost between the RP2.1 + test scenario and current care scenario was assumed to be the cost savings associated with AMR that is attributable to the RP 2.1 + test. The resulting cost savings was then adjusted to obtain an annual cost value. Different scenarios were also explored including the application of other costs of AMR. An ongoing study by Naylor et al. 2024 [40] also estimated the global hospital cost of and productivity loss associated with ABR to be $693 (£554) and $194 (£155) billion annually. The RMf was not applied to the costs obtained from the Naylor study since these costs were associated with the human population. We only applied the assumption that COPD associated costs is 7.5% of the resulting costs. The resulting costs were then assumed to be the cost savings associated with the RP 2.1 + test.

In a final analysis, we sought to look at the potential savings associated with adopting the RP 2.1 + test in the UK. For the modelling exercise, the unit cost associated with the RP 2.1 + test was £94 per patient. In the UK, around 1.4 million people have a diagnosis of COPD and the yearly diagnosis is about 115,000 [41]. A recent study estimated that between 30% and 50% of COPD patients have at least one exacerbation per year [42]. Thus, the number of patients with exacerbations are estimated to be 34,500 and 57,500 respectively if 30% and 50% of COPD patients have exacerbations. The follow on analysis suggests that on an annual basis at a cost of £94 per patient, the costs of using the RP 2.1 + tests could range from £3.2 million to £5.4 million if 30% and 50% of COPD cases are tested. We tested scenarios where the cost savings associated to the RP 2.1 + test that can be attributable to the UK were assumed to be 50% and 25% of the cost savings that were obtained from the O’Neil study [43]. This costs was then compared the costs of administering the RP 2.1 + test to all COPD patients who have an exacerbation in the UK.

## Results

**Fig 1:**
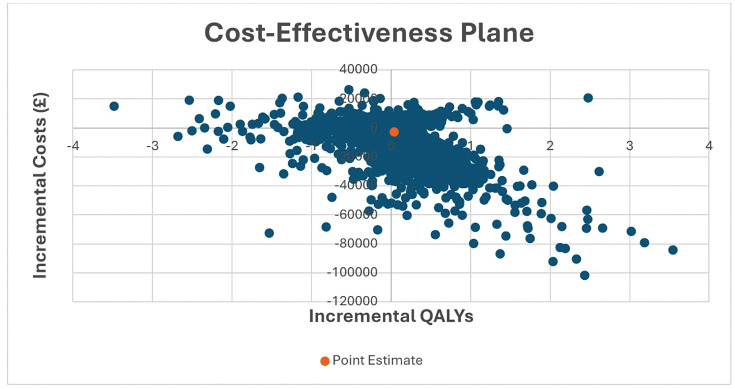
Cost-effectiveness plane.

**Fig 2:**
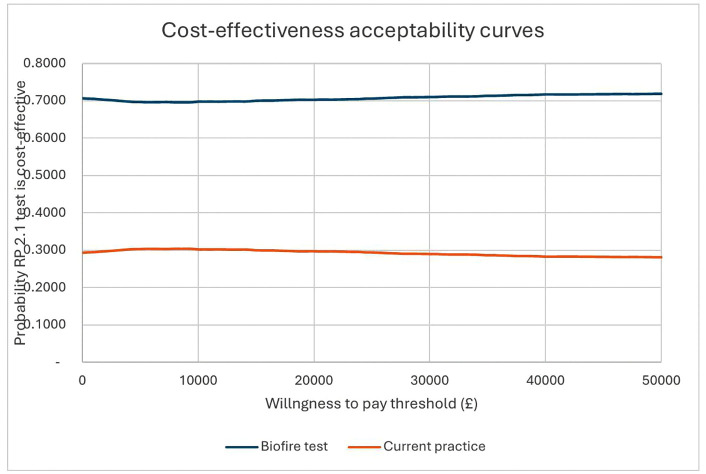
Cost-effectiveness acceptability curve.

The results indicates that the RP 2.1 + test is more effective in terms of QALYs and less costly than usual practice. The cost and QALY difference between the RP 2.1 + test and no test arm were £2762.40 and 0.03 respectively (Table 5) and suggesting that the RP 2.1 + test is cost-effective at the National Institute for Health and Care Excellence (NICE) threshold of £20,000–30,000 per QALY gained. Incremental costs and outcomes and the cost-effectiveness acceptability curve has been presented in Fig 1 and Fig 2 below. This shows that at the NICE threshold of £20,000 per QALY gained, there is a 70% chance that the RP 2.1 + test is cost-effective. The one-way sensitivity analysis seems to suggest that in all scenarios, the RP 2.1 + test was cost-effective (Table 6). Threshold analysis revealed that the RP 2.1 + test would not be cost-effective if the cost of the RP 2.1 + test exceeds £355 per unit.

**Table 5:** Results Base Case.

	Costs (£)	QALYs
**RP2.1 test**	57,340.81	9.747
**Usual practice**	60,103.22	9.721
**Difference**	−2762.40	0.03
**ICER**	RP2.1 test dominant	

**Table 6:** DSA results (Short-Run).

Costs	Base case parameter value	Minimum parameter value	ICER	Maximum parameter value	ICER
RP2.1 test	£94	£57	RP 2.1 + test dominant	£131	RP 2.1 + test dominant
A&E without admission	£499	£303	RP 2.1 + test dominant	£695	RP 2.1 + test dominant
COPD hospital stay	£1,930	£1,173	RP 2.1 + test dominant	£2,687	RP 2.1 + test dominant
Antibiotic course	£1.84	£1.12	RP 2.1 + test dominant	£2.56	RP 2.1 + test dominant
**Utilities**					
Partial recover	0.56	0.34	RP 2.1 + test dominant	0.77	RP 2.1 + test dominant
Full recover	0.66	0.40	RP 2.1 + test dominant	0.92	RP 2.1 + test dominant
**Probabilities**					
**Control arm**					
Antibiotics after initial COPD	0.8	0.49	RP 2.1 + test dominant	1.00	RP 2.1 + test dominant
Hospitalisation if patient had bacterial infection	0.20	0.12	RP 2.1 + test dominant	0.28	RP 2.1 + test dominant
Hospitalisation if patient had viral infection	0.10	0.06	RP 2.1 + test dominant	0.14	RP 2.1 + test dominant
**Intervention arm**					
Antibiotics after initial COPD	0.03	0.02	RP 2.1 + test dominant	0.04	RP 2.1 + test dominant
Hospitalisation if patient was initially treated by ab	0.15	0.09	RP 2.1 + test dominant	0.21	RP 2.1 + test dominant
Hospitalisation if patient was initially treated by st and ab	0.10	0.06	RP 2.1 + test dominant	0.14	RP 2.1 + test dominant

### Accounting for the cost of AMR

With approach 1, the RP 2.1 + test was dominant irrespective of the costs of AMR that had been applied. (Table 7).

**Table 7:** Accounting for the Cost of AMR (Approach 1).

		Cost of AMR	ICER
**O’Neil et al. 2014**		£105.76^a^	RP 2.1 + test dominant
**Shrestha et al. 2016**	**Broad Spectrum**	£7.45^b^	RP 2.1 + test dominant
	**Narrow Spectrum**	£14.89^b^	RP 2.1 + test dominant

^**a**^ Includes societal costs ^**b**^ Does not include wider societal costs.

**Fig 3:**
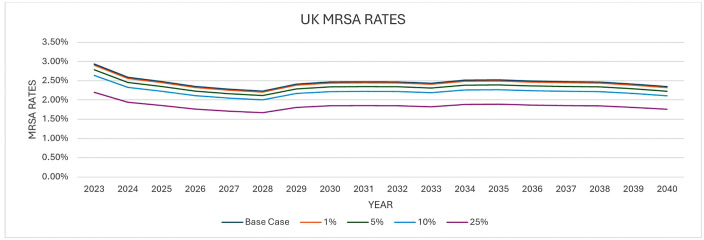
AMR Predictions (MRSA Rates).

**Fig 4:**
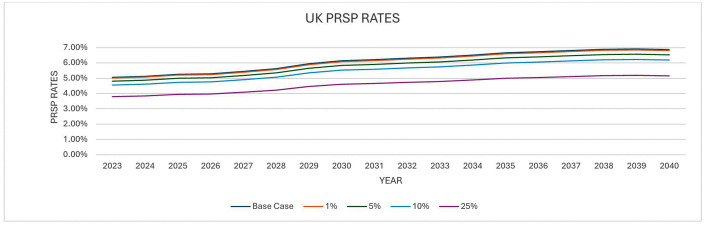
AMR Predictions (PRSP Rates).

With approach 2, the United Kingdom AMR predictions for Methicillin-resistant *Staphylococcus aureus* (MRSA) and Penicillin-resistant *Streptococcus pneumonia* (PRSP) between 2003 and 2040 are presented in Fig 3 and Fig 4. These present a scenario where AMR develops as usual versus a scenario where AMR is reduced because of the use of the RP 2.1 + test.

For MRSA, the projections from the study indicate that the annual cost savings because of the RP 2.1 + test could range from £529 million to £12.6 billion in terms of GDP savings. Annual hospital cost savings range from £353 million to £8.65 billion whilst annual productivity cost savings range from £50 million to £2.3 billion. Combined productivity costs and hospital cost savings ranged from £403 million to £11 billion (Table 8). Assuming the costs in the UK is as low as 50% of the costs, the resulting savings ranges from 252 million to 6.3 billion annually. If costs are assumed to be as low as 25% of AMR cost, the resulting cost savings ranges from 118 million to 2.9 billion (Table 8).

**Table 8:** MRSA costs savings from adopting RP 2.1 + TEST (£) (Approach 2).

	1%	5%	10%	25%
	RP 2.1 + test	RP 2.1 + test	RP 2.1 + test	RP 2.1 + test
**2.2 Trillion**				
Annual Cost savings	529 million	2.5 billion	5.1 billion	12.6 billion
Annual Cost Savings if UK costs = 50%	252 million	1.2 billion	2.4 billion	6.3 billion
Annual Cost Savings if UK costs = 25%	118 million	647 million	1.3 billion	2.9 billion
	**£554 Billion Hospital Costs**			
Annual Cost savings	353 million	1.7 billion	3.4 billion	8.65 billion
	**£155 Billion Productivity Costs**			
Annual Cost savings	50 million	470 million	941 million	2.3 billion
	**Hospital plus Productivity Costs**			
Total Cost Savings (Hospital & Productivity)	403 million	2.1 billion	4.3 billion	11 billion

For PRSP, the model indicates that the cost savings from adopting a test like this could range from £741 million to £18.6 billion annually in terms of GDP savings. Hospital cost savings range from £470 million to £12.6 billion annually. Productivity costs savings annually ranged from £118 million to 3.5 billion. Combined annual costs savings ranged from £588 million to £16.1 billion (Table 9). Assuming the UK costs of AMR were 50%, the resultant cost savings would range from 353 million annually to 88 billion annually. If UK costs were as low as 25% of the costs, the resulting cost savings would range from £176 million to 4.6 billion annually (Table 9).

**Table 9:** PRSP costs savings from adopting RP 2.1 + TEST (£) (Approach 2).

	1%	5%	10%	25%
	RP 2.1 + test	RP 2.1 + test	RP 2.1 + test	RP 2.1 + test
**2.2 Trillion**				
Annual Cost savings	741 million	3.7 billion	7.4 billion	18.6 billion
Annual Cost Savings if UK costs = 50%	353 million	1.8 billion	3.5 billion	8.8 billion
Annual Cost Savings if UK costs = 25%	176 million	882 million	1.9 billion	4.6 billion
	**554 Billion Hospital Costs**			
Annual Cost savings	470 million	2.5 billion	5.1 billon	12.6 billion
Annual Cost savings	118 million	706 million	1.4 billion	3.5 billion
	**155 Billion Productivity Costs** **Hospital plus Productivity Costs**			
Total Cost Savings (Hospital & Productivity)	588 million	3.2 billion	6.5 billion	16.1 billion

The prediction also suggests that the annual UK costs of using the RP 2.1 + tests could range from £3.2 million to £5.4 million if 30% and 50% of COPD cases are tested annually. These costs are still far lower than the AMR cost savings that would accrue if the UK costs are 50% and 25% lower than global costs for both the case of MRSA and PRSP (Tables 8–9).

## Discussion

The results of the study indicate that the RP 2.1 + test is dominant over current practice, i.e., more effective in terms of QALYs and less costly than current practice. At a £20,000 per QALY threshold, there is a 70% chance that the test is cost-effective. Threshold analysis indicates that only if the RP 2.1 + test’s cost exceeds £355 per patient would it cease to be cost-effective. The study aimed to account for the cost of AMR and the potential impact of the RP 2.1 + test. As expected, the RP 2.1 + test remained dominant or cost-saving after various penalty points were added to antibiotic prescriptions. In terms of potential AMR additional cost savings, the analysis showed that annually, this could range between £529 million and £18.6 billion in terms of GDP savings and range between £403 million and £16.1 billion in terms of hospital costs and productivity losses. The potential cost savings with respect to AMR could range between £118 million and £8.8 billion if the cost of AMR in the UK was assumed to range between 25% and 50% of estimated costs. When compared to the maximum costs required to test all patients in the UK, the cost savings was still significant.

This study is associated with several strengths. First, this is the first study to assess the cost-effectiveness of the RP 2.1 + test in patients with exacerbations of COPD in the community. Second, to the best of our knowledge, this is the first study to also account for the cost of AMR within economic evaluations in this patient group and look at potential savings that could be accrued in terms of AMR.

To model the implications of AMR, several simplifying assumptions were made. First, although there is an association between antibiotic consumption and the development of AMR, there is little consensus with respect to this, we therefore made a conservative assumption that the reductions in the rates of AMR from reductions in antibiotic consumption could range between 1% and 25%. It is also important to point out that the relationship could vary over time. However, due to lack of evidence in this respect, we assumed that this relationship is constant over time. Second, in our attempt to apply the cost of AMR to the UK, we assumed that the UK costs are similar to the global costs. We however carried out sensitivity analysis where we assumed the UK costs could be between 25% and 50% of the global costs. The results from this assumption led to similar results which showed that there is a significant cost saving. Third, we only considered the case of MRSA and PRSP as proxies for AMR due to the availability of data. However, we acknowledge that COPD exacerbations and respiratory infections may involve a wider spectrum of resistant bacteria; therefore, the AMR-related estimates should be interpreted as exploratory and not as a complete representation of the total resistant pathogen burden in this population.

A recent study by Abel et al [18], assessing a similar diagnostic test in this patient group also established cost-effectiveness of the diagnostic test. This study indicated that the test would need to be greater £260 for it not to be cost-effective. Both findings are like what we found in our study. Our study found the RP 2.1 + test to dominant (cost saving and effective) and found that the test would need to cost as much as £355 for it not to be cost-effective.

The results of this study have highlighted the dominance of the RP 2.1 + test in patients undergoing treatment for exacerbations of COPD and shown the potential benefits and cost savings from reduced expenditure on AMR that would occur if the RP 2.1 + test is adopted. In addition, the finding that the potential cost savings from reductions in AMR which outweighs the investments needed to adopt the RP 2.1 + test would be encouraging to decision/policy makers who are already tasked with allocating scarce healthcare resources. Identifying infections early on would also lead to appropriate action in terms of better targeting of treatments and other actions which would reduce the economic burden that is associated with exacerbations of COPD.

Our study further highlights the need to account for the costs of AMR within economic evaluations. However, there is still little consensus with respect to the appropriate methodology to achieve this accurately. The very wide range in the annual potential cost savings obtained in this study reflects the uncertainty associated with accounting for AMR within economic evaluation studies. One of the several issues that was highlighted in this study is the relationship between antimicrobial resistance and antibiotic consumption/prescribing. The exact elasticities between antibiotic consumption and antimicrobial resistance is uncertain. Therefore, future research should focus on estimating these elasticities based on available data on consumption and AMR.

## Supporting information

S1 Fig Decision tree pathways (Biofire arm). Ab antibiotics, st stands for steroids, and AE stands for accidents and emergencies.
(DOCX)

S2 Fig Decision tree pathways (Current practice arm). (DOCX)

S3 Fig Markov Model adapted from Abel et al (2019). (DOCX)

S1 File PSA data. (XLSX)
