# Supplementary material for: Cost-effectiveness of biofire assay for respiratory infection testing: An economic evaluation exploring the inclusion of the costs of antimirobial resistance
Source: PLoS One. 2026 Apr 27;21(4):e0347991. doi: 10.1371/journal.pone.0347991 (PMC13119865; doi:10.1371/journal.pone.0347991)
Supplement: S2 Fig — (DOCX) [file pone.0347991.s002.docx]

|  |  | Improve |  |  |  |  |  |  |  |
| --- | --- | --- | --- | --- | --- | --- | --- | --- | --- |
|  |  | 0.80 |  |  |  | full recover |  |  |  |
|  | Treat |  | Improve |  |  |  |  |  |  |
|  | Ab |  | 0.87 |  |  |  |  |  |  |
|  | 0.80 | Deteriorate (St + ab, or AE) |  |  |  |  | Full recover |  |  |
|  |  | 0.20 |  |  | Improve |  | 0.89 |  |  |
|  |  |  |  |  | 0.80 |  | Partial recover |  |  |
|  |  |  | Deteriorate (2nd ab) |  |  |  | 0.11 |  | Full recover |
|  |  |  | 0.13 |  |  |  | Improve |  | 0.89 |
| Current practice |  |  |  |  |  |  | 0.95 |  | Partial recover |
|  |  |  |  |  | Hospital | |  |  | 0.11 |
|  |  |  |  |  | 0.20 |  | Dead |  |  |
|  |  | Improve |  |  |  |  | 0.05 |  |  |
|  |  | 0.89 |  |  |  |  | full recover |  |  |
|  | Treat (st +ab) |  | Improve | Improve |  |  |  |  |  |
|  | 0.20 |  | 0.80 |  |  | Full recover |  |  |  |
|  |  | Deteriorate (St + ab, or AE) |  | Improve |  | 0.89 |  |  |  |
|  |  | 0.12 |  | 0.90 |  | Partial recover |  |  |  |
|  |  |  | Deteriorate (st + ab or AE) |  |  | 0.11 |  | Full recover |  |
|  |  |  | 0.20 |  |  | Improve | | 0.89 |  |
|  |  |  |  |  |  | 0.95 |  | partial recover |  |
|  |  |  |  | Hospital |  |  |  | 0.11 |  |
|  |  |  |  | 0.10 |  | Dead |  |  |  |

S2 Fig. Decision tree pathways (Current practice arm)
