# Supplementary material for: Cost-effectiveness of biofire assay for respiratory infection testing: An economic evaluation exploring the inclusion of the costs of antimirobial resistance
Source: PLoS One. 2026 Apr 27;21(4):e0347991. doi: 10.1371/journal.pone.0347991 (PMC13119865; doi:10.1371/journal.pone.0347991)
Supplement: S3 Fig — (DOCX) [file pone.0347991.s003.docx]

COPD exacerbation

COPD exacerbation

COPD exacerbation

S3 Fig: Markov Model adapted from Abel et al (2019)
